# Sticky decisions: The multilayered regulation of adhesin production by bacteria

**DOI:** 10.1371/journal.pgen.1010648

**Published:** 2023-03-02

**Authors:** Cecile Berne

**Affiliations:** Département de microbiologie, infectiologie et immunologie, Université de Montréal, Montréal, Québec, Canada; Indiana University Bloomington, UNITED STATES

Biofilms are communities of microorganisms attached to a surface. The biofilm is a lifestyle that provides numerous advantages to its bacterial inhabitants, as it can offer shelter from predators, favor exchange of nutrients and genetic material, and provide resistance to xenobiotic stresses. However, conditions around or within the biofilm can deteriorate and bacteria may need to escape to avoid a death threat. Consequently, to stick or not to stick to a biofilm is a crucial decision. The fate of an individual bacterium often relies on its ability to either adhere and settle in an environment or disperse. Likewise, the fate of the entire colony often relies on managing its population as a function of environmental conditions. For these reasons, adhesion is usually the result of combined actions of several tightly regulated processes. A recent *PLOS Genetics* paper by Maeve McLaughlin and colleagues describes a new transcription factor involved in the regulation of bacterial adhesion and sheds light on the complexity and multifactorial nature of this regulation [1].

There are multiple stages in the making of a biofilm [2]. Swimming or free-floating bacteria in the planktonic phase first approach a surface and interact with it thanks to various extracellular appendages, such as flagella and pili. This initial adhesion is reversible and cells can still leave the surface if conditions are not optimal. However, if the environment is favorable, these attached cells can commit to the biofilm lifestyle by strengthening their adhesion to the surface and become irreversibly attached. Obviously, the processes that govern transitions between motile and sessile lifestyles, reversible and irreversible adhesion, must be tightly regulated. Once permanent adhesion is achieved, bacteria attached to the surface multiply, the biofilm grows, and two fates are possible for the newborns: join the biofilm or leave the biofilm and disperse. Bacteria that stay in the biofilm have developed several, nonexclusive strategies to ensure strong permanent adhesion. Some bacteria interact with the surface thanks to adhesins located around their cell body, such as fimbriae and other adhesin proteins. Other bacteria secrete an extracellular matrix, usually composed of polysaccharides, proteins, and / or DNA molecules which help trap cells close to the surface. Finally, most Alphaproteobacteria rely on a strong polar polysaccharide adhesin to irreversibly attach to the surface [3]. The holdfast of *Caulobacter crescentus* is the best characterized example of such polarly located adhesins, and this polysaccharide is itself responsible for irreversible adhesion and biofilm formation.

*C*. *crescentus* has a dimorphic lifecycle where each motile newborn cell (swarmer) bears a flagellum and several pili at one pole. Upon transition to the sessile form, the swarmer cell sheds its flagellum and retracts its pili, entering the non-motile phase of the lifecycle. An adhesive holdfast is synthesized at the pole previously bearing flagellum and pili. Then the cell produces a polar stalk that pushes the holdfast away from the cell body. The resulting stalked cell elongates into a predivisional cell and synthesizes a new flagellum at the pole opposite the stalk. Finally, each predivisional cell divides and gives birth to a new motile swarmer cell.

Holdfast production is tightly regulated by several independent mechanisms controlled at numerous stages during the lifecycle of the bacterium. This multilayer mechanism ensures that holdfast is produced in a timely manner during the cell cycle and only when conditions are favorable. Indeed, as holdfast-mediated adhesion is irreversible, it is important that holdfast is produced only when the environment is suitable for reproduction, development, and habitat colonization. Holdfast is produced via two distinct pathways, in the presence or in the absence of a surface. Conditions permitting, holdfast is synthesized within seconds upon contact after *C*. *crescentus* encounters a surface [2,4]. Surface sensing and subsequent holdfast production result from the combined action of the flagellum and pilus machineries [4–6]. The first interaction between the pili and the surface creates a resistance for their retraction, which consequently stimulates the production of the nucleotide second messenger molecule cyclic di-GMP (cdG) by the diguanylate cyclase PleD [7,8]. This generates the first cue which eventually stimulates holdfast production [4]. cdG production induced by hampered pili retraction also triggers cell differentiation [7,8], stimulating the formation of holdfast-bearing stalked cells that will attach irreversibly to the surface. Simultaneously, pili retraction also brings the polar flagellar machinery, located at the same pole of the cell, in contact with the surface. This event triggers the second signal for holdfast production [5,6]. While the flagellum filament or its rotation are dispensable for the surface contact response [5,9], the motor senses the surface upon direct contact via an unknown mechanism involving proton motive force and intracellular pH changes [5]. Upon contact, the flagellar motor triggers the production of cdG via the diguanylate cyclase DgcB. The produced cdG binds to the predicted glycolipid glycosyltransferase HfsJ, leading to the activation of this protein crucial for holdfast synthesis [5].

**Fig 1:**
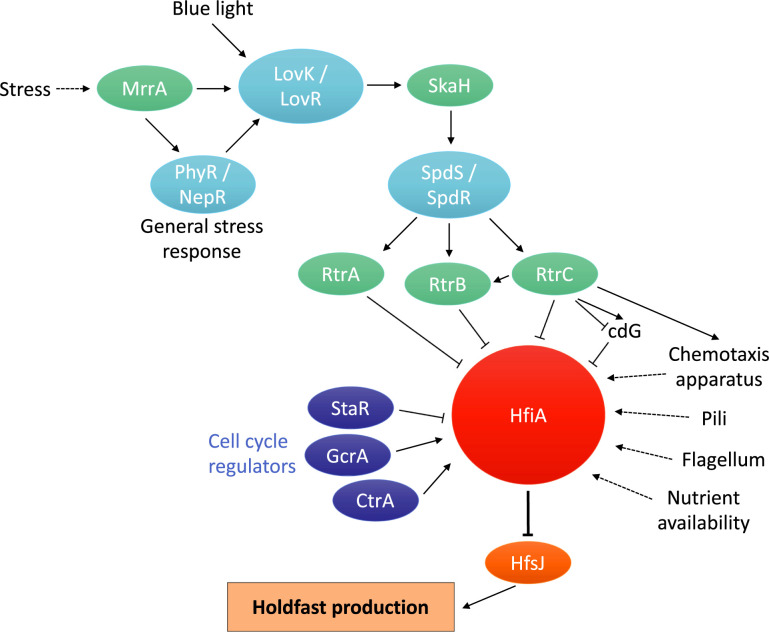
HfiA is a master regulator of holdfast production and irreversible adhesion in *C*. *crescentus*. The small protein HfiA (Holdfast inhibitor A) is regulated at the transcriptional level by several players as described in the main text.

*C*. *crescentus* does not only produce holdfast upon contact with a surface, but it can also synthesize a holdfast in the absence of a surface. In that case, holdfast production is part of a complex developmental program that leads newborn swarmer cells to differentiate into stalked cells. Holdfast production during the cell cycle is regulated by levels of cdG inside the cell [10,11]. cdG is an important player in the switch from motile to sessile lifestyles in many bacteria [12] and has been shown to be crucial for proper timing of holdfast synthesis upon cell differentiation: holdfast synthesis is temporally regulated during the cell cycle and holdfast is produced at the pole when cells reach late swarmer cell stage [10]. The main regulator of holdfast synthesis in the absence of a surface is the holdfast inhibitor protein HfiA [13]. HfiA directly interacts with HfsJ, a protein crucial for holdfast synthesis [13]. As depicted in Fig 1, HfiA itself is subjected to a complex multi-layered regulation. An ever-growing set of regulators act in concert and maintain HfiA at levels which enable proper holdfast synthesis when conditions and timing are suitable. First, the cell cycle regulators StaR, GcrA, and CtrA bind to the *hfiA* promoter and control its expression, thus ensuring the timing of holdfast synthesis during cell cycle differentiation [13]. Another layer of regulation occurs in response to stressful environmental signals such as nutrient limitation [13] and the general stress response [14], via the action of two-component systems (TCS) such as LovK / LovR [15] and the single-domain response regulator MrrA [16]. Both LovK / LovR and MrrA control *hfiA* transcription [13,16] via a complex network of TCS including the hybrid histidine kinase SkaH, the TCS SpdS / SpdR, and the RegBA transcriptional regulators RtrA and RtrB [17]. Furthermore, *hfiA* expression is also indirectly regulated by flagellum and pili assembly and by chemotaxis proteins by yet unknown mechanisms [9,18,19] (Fig 1). Finally, the chaperone DnaK is crucial for HfiA stabilization in the cell once the protein is synthesized [20].

A few years ago, David Hershey and the Crosson laboratory designed a clever unbiased genome-wide screen using a barcoded transposon library in *C*. *crescentus* where mutants impaired in adhesion were enriched by their inability to bind to cheesecloth. Adhering bacteria efficiently bind to the cheesecloth, therefore leaving the liquid medium enriched with non-adhering bacteria. The transposon library was grown in the presence of cheesecloth for multiple days, and each day, the medium containing unattached cells was used to inoculate a fresh culture containing a sterile cheesecloth piece [18]. This simple enrichment was instrumental for important findings in holdfast regulation. For example, it helped determine that motility, flagellum synthesis, and type IV pili assembly are involved in adhesion via holdfast production by regulating *hfiA* expression [18]. More recently, this screen led to the characterization of the flagellar signaling suppressor (*fss*) genes which also contribute to holdfast regulation by acting downstream of flagellum assembly to regulate *hfiA* expression [21]. That same work also showed that improper flagellum assembly regulates holdfast production via two distinct pathways: a ’mechanical pathway’ regulated by load on the flagellar filament via the stator proteins and the diguanylate cyclase DgcB and a ’developmental pathway’ regulated by the presence of a functional flagellum, the *fss* genes, and the diguanylate cyclase PleD [21]. Both pathways converge to regulate *hfiA* transcription using different routes.

McLaughlin and colleagues performed a similar cheesecloth enrichment using a hyper-adhesive strain that overexpresses a non-phosphorylatable LovK (*lovK*_H180A_) mutant leading to the misregulation of *hfiA* expression and overproduction of holdfasts [17]. Two transcription factors, RtrA and RtrB, were previously shown to directly bind to the *hfiA* promoter and repress its transcription [17]. The work by McLaughlin and colleagues highlights a new transcription factor involved in this pathway, RtrC. The latter binds a pseudo-palindromic motif present in the *hfiA* promoter region and represses its expression [1]. All the work accumulated by the Crosson laboratory over the years places HfiA as a key player in holdfast regulation (Fig 1). Changes in *hfiA* transcription cause changes in holdfast production and subsequent cell attachment, so it is not surprising to find out that the regulation of this master regulator of holdfast synthesis is finely tuned by a complex network that acts upon different conditions and at different levels.

**Fig 2:**
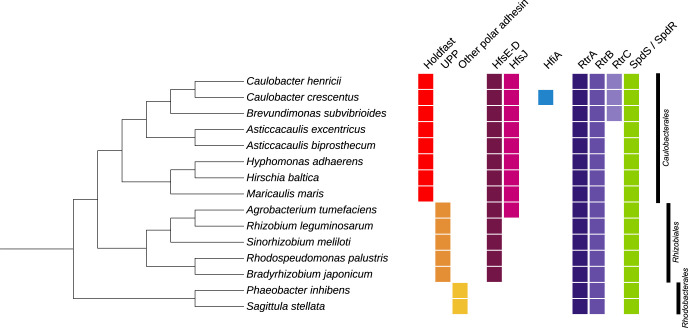
Conservation of proteins involved in holdfast regulation via HfiA in *C*. *crescentus* among several Alphaproteobacteria bearing a polar adhesin. Several species reported in the literature as bearing a polar adhesin have been selected to build this phylogenetic tree: *Caulobacter crescentus* [23] and *C*. *henricii* [45], *Brevundimonas subvibrioides* [45], *Asticcacaulis excentricus* [23] and *A*. *biprosthecum* [46], *Hyphomonas adhaerens* [47], *Hirschia baltica* [26], *Maricaulis maris* [45], *Agrobacterium tumefaciens* [48], *Rhizobium leguminosarum* [39], *Sinorhizobium meliloti* [49], *Rhodospeudomonas palustris* [27], *Bradyrhizobium japonicum* [50], *Phaeobacter inhibens* [29] and *Sagittula stellata* [30]. The tree was built using a concatenated alignment of 7 conserved proteins (FusA, GyrA, GyrB, RecA, RpoA, and RpoB) aligned using MUSCLE [51] and constructed using iTOL [52]. Red, orange et yellow boxes represent the presence of a polar adhesin (holdfast, UPP, or other respectively). The presence of homologous proteins was determined by BLAST [53] reciprocal best hits, with cut-offs of E value > 10^−5^ and sequence identity > 50%. The presence of homologs for the HfsE-D holdfast cluster, HfsJ, HfiA, RtrA, RtrB, RtrC, and SpdS / SdpR is represented by a colored box. The absence of the box indicates that no protein satisfied the BLAST criteria.

Using a polar adhesin to bind to surfaces is a trait shared by many Alphaproteobacteria [3,22] (Fig 2). Polar adhesins are involved in binding to surfaces, but also in forming polarly arranged cell aggregates called rosettes [23]. So far, only two types of these polar adhesins have been extensively characterized: the holdfast in the *Caulobacterales* [23,24] and the unipolar polysaccharide UPP in the *Rhizobiales* [25]. The holdfast synthesis gene cluster (*hfsEFGHCBAD*) is found in *Caulobacterales* that synthesize a holdfast [24,26], while the *uppABCDEF* UPP cluster is conserved in the *Rhizobiales* [27,28] (Fig 2). Other Alphaproteobacteria have also been reported to attach to surfaces using a polar adhesin and / or form characteristic rosettes, such as the *Rhodobacterales Phaeobacter inhibens* [29], *Sagittula stellata* [30] and some marine *Roseobacters* [31]. However, little is known about the synthesis and composition of those polar adhesins, and these bacteria do not have homologs in either of the *hfsE-D* or *uppA-F* clusters (Fig 2).

In addition to the synthesis clusters mentioned above, a series of other proteins are essential for holdfast and UPP formation, such as glycosyltransferases (HfsJ and HfsI in *C*. *crescentus* [32] and *H*. *baltica* [26], or UppL in *A*. *tumefaciens* [28]) (Fig 2). While homologs of HfsJ are conserved in the *Caulobacterales*, UppL is present only in several *A*. *tumefaciens* strains [28] and no homologs could be found in other *Rhizobiales* and *Rhodobacterales* (Fig 2). The Crosson laboratory showed previously that, in *C*. *crescentus*, HfsJ activity is inhibited by direct interaction with HfiA, preventing holdfast to be synthesized [13]. Interestingly, there are no known homologs of HfiA in other species than *C*. *crescentus* (Fig 2). In addition, while RtrA and RtrB are widely spread in the Alphaproteobacteria, RtrC homologs can only be found in *Caulobacterales* closely related to *C*. *crescentus* [1] (Fig 2). But while HfiA is unique *to C*. *crescentus*, RtrC (directly controlling HfiA expression) is restricted to closely-related species, and HfsJ (HfiA direct target) is not present in all Alphabacteria bearing a polar adhesin, the *hfiA* regulators LovK / LovR and SpdS / SpdR are not only found in most Alphaproteobacteria but also widespread among other bacteria (Fig 2) [33,34].

The LovK / LovR TCS are LOV (light, oxygen, voltage) blue light photoreceptor proteins conserved in bacteria, archaea, plants, and fungi [33]. While photoreceptors were first thought to be crucial only for photosynthetic organisms that use sunlight as an energy source, we now know that non-phototrophic bacteria also respond to light to regulate important lifestyle decisions such as motility, virulence and adhesion [33,35]. In addition to *C*. *crescentus* holdfast regulation, LOV proteins have been shown to play a role in regulating adhesion in *Xanthomonas axonopodis* [36], *Ralstonia pseudosolanacearum* [37], and *Rhizobium leguminosarum* where it regulates extracellular exopolysaccharide production [38]. Interestingly, *R*. *leguminosarum* produces a UPP polar polysaccharide [39] (Fig 2) and it would be interesting to know if its production is also regulated by light via the LOV complex. Homologs of the SpdS / SpdR TCS are also present in many species, although under a confusing plethora of names [34]. These key TCS are major regulators of photosynthesis and other metabolic processes, but are also involved in the regulation of many other cellular processes [40]. They have been reported in various species to control motility and / or adhesion. In *Rhodobacter capsulatus*, the RegB / RegA regulon controls the majority of genes involved in motility [41] and, in *Rhodobacter sphaeroides*, PrrB / PrrA regulates aerotaxis [42]. Whereas, in *Pseudomonas aeruginosa*, RoxS / RoxR regulates bacterial attachment to epithelial cells [43], the MSMEG_0244 / MSMEG_0246 and PrrB / PrrA pairs control biofilm formation in *Mycobacterium smegmatis* and *R*. *sphaeroides* respectively [43,44]. In *C*. *crescentus*, SpdS / SpdR controls the expression of at least three *hfiA* transcriptional regulators: RtrA, RtrB [17], and the newly described RtrC [1]. These regulators modulate holdfast production and biofilm formation. Interestingly, the work by McLaughlin *et al*. shows that, in *C*. *crescentus*, RtrC can act as a repressor or activator of gene expression depending on its binding site location [1]. In addition to the *hfiA* promoter, RtrC binds to the promoter of genes involved in cdG signaling, motility, and chemotaxis [1]. This suggests that the entire pathway might regulate more than irreversible adhesion via HfiA-dependent holdfast production. This work provides hints for RtrC acting as a regulator operating at the crossroad of opposing lifestyles: sessile or motile. It will be interesting to determine whether this complex and multi-layered regulation cascade could also be involved in other steps of the motile to sessile transition. This transition must also be tightly controlled in other bacteria, and little is known about the regulators involved in this switch in other Alphaproteobacteria [25]. As discussed above, RtrC homologs are present only in species closely related to *C*. *crescentus* [1] (Fig 2), and more studies are needed to determine how unique this regulation system is and how other bacteria producing a polar adhesin switch from motile to sessile lifestyles.

In conclusion, the work by McLaughlin *et al*. adds a new player involved in the complex regulation cascade of HfiA, illustrating that this small protein acts as a master regulator of holdfast production and irreversible adhesion in *C*. *crescentus* [1]. HfiA and RtrC are unique to close relatives of *C*. *crescentus*. It would also be interesting to know how other species that produce polar polysaccharides to irreversibly adhere to surfaces and form biofilms ensure proper timing of synthesis of their adhesins when conditions are favorable. Do they also rely on RegB / RegA or other TCS? Do they have several regulation pathways and / or a master regulator like HfiA, controlled at multiple levels?
